# Resistance Training Associated with Dietetic Advice Reduces Inflammatory Biomarkers in the Elderly

**DOI:** 10.1155/2020/7351716

**Published:** 2020-09-04

**Authors:** Lilian Maria Peixoto Lopes, Emerson Cruz de Oliveira, Lenice Kappes Becker, Guilherme de Paula Costa, Kelerson Mauro de Castro Pinto, André Talvani, Júlia Cristina Cardoso Carraro, Daniel Barbosa Coelho

**Affiliations:** ^1^Postgraduate Program in Health and Nutrition, Federal University of Ouro Preto, Ouro Preto, Minas Gerais, Brazil; ^2^Physical Education School, Federal University of Ouro Preto, Ouro Preto, Minas Gerais, Brazil; ^3^Inflammation Immunobiology Laboratory, Federal University of Ouro Preto, Ouro Preto, Minas Gerais, Brazil; ^4^School of Nutrition, Federal University of Ouro Preto, Ouro Preto, Minas Gerais, Brazil

## Abstract

Aging is a biological process during which chronic low-grade inflammation is present due to changes in the immune system of the elderly. The main objective of this study is to evaluate the effects of resistance training associated with dietary advice on chronic inflammation in the elderly. We conducted a prospective intervention study in which we evaluated anthropometric parameters and inflammatory biomarkers (CRP, IL-8, CCL-2, and leptin) in 40 elderly people before and after long-term progressive resistance training (19 weeks) associated with dietary advice. The participants trained twice a week on nonconsecutive days, and the training lasted one hour with an intensity of 60-85% of 1-MR. Dietary advice was explained in person and individually focusing on foods rich in compounds with anti-inflammatory and antioxidant properties. Participants were instructed at the beginning of the training program, and dietary advice was reinforced verbally weekly. There was an improvement in body composition evidenced by a reduction in waist circumference and body fat percentage and by the increase in arm circumference, calf circumference, and corrected arm muscle area. In addition, there was a reduction in the inflammatory biomarkers CCL-2 (*p* = 0.01) and leptin (*p* < 0.01). Resistance training associated with dietary guidance can contribute to a healthy aging due to observed improvements in body composition and in the inflammatory profile of the elderly.

## 1. Introduction

The growing number of elderly people in Brazil highlights the peculiarities of this population, which interfere with the social and economic context. Factors such as the presence of mostly chronic diseases and morphological, physiological, and body composition changes tend to affect the nutritional status and the health of the elderly [1–4].

Chronic low-grade inflammation is common in the elderly due to changes in the immune system that lead to dysregulation in cytokine secretion. In addition, as a consequence of changes in body composition, the secretion of proinflammatory cytokines by adipose tissue increases [5–9].

A pertinent issue to be addressed in reducing low-grade chronic inflammation is resistance training. Resistance exercise generates adaptations in the skeletal muscle. It reduces the secretion of proinflammatory cytokines and increases the expression of anti-inflammatory cytokines. In addition, this type of training can decrease adiposity and increase the activity and expression of antioxidant enzymes [10–14].

Still regarding low-grade chronic inflammation, other studies have shown that reductions in the consumption of saturated fats and simple sugars and increases in the consumption of foods rich in compounds with anti-inflammatory and antioxidant action (such as polyphenols, prebiotics, and omega 3 fatty acids) tend to decrease plasma concentrations of inflammatory biomarkers in the elderly [15–17].

However, the few existing studies on resistance training related to chronic inflammation in the elderly have addressed physical exercise in isolation, without taking into account the possible synergistic effects of dietary advice focusing on foods with anti-inflammatory and antioxidant properties [18–21, 4, 22]. Thus, this study proposes to investigate the effects of resistance training associated with dietary advice focusing on foods rich in compounds with anti-inflammatory and antioxidant actions on low-grade chronic inflammation in the elderly.

## 2. Materials and Methods

### 2.1. Ethical Aspects

This study was conducted according to the guidelines of the Resolution of the National Health Council no. 466/2012. It was approved by the Research Ethics Committee on Humans of the Federal University of Ouro Preto (CAAE: 02761918.0.0000.5150). Participants were duly informed about the risks and benefits and were included in the study after signing an informed consent.

### 2.2. Study Design, Selection of Participants, and Eligibility Criteria

This is a prospective study. We evaluated the following anthropometric parameters: weight, body mass index (BMI), waist circumference (WC), hip circumference (HC), waist-hip ratio (WHR), abdominal circumference (AC), arm circumference (ArmC), calf circumference (CC), and corrected arm muscle area (AMAc). We also evaluated inflammatory biomarkers (CRP, IL-8, CCL-2, and leptin) before and after the long-term progressive resistance training intervention and nutritional advice for 19 weeks.

Participants were recruited through posters posted at the Federal University of Ouro Preto (UFOP) and throughout the community of Ouro Preto. Interested participants performed a face-to-face registration. They were later scheduled for the initial assessment by telephone.

The inclusion criteria were age equal to or above 60 years and absence of problems that prevented physical activity (resistance training). A specialized professional evaluated them. The exclusion criterion was a percentage of attendance in the training program lower than 70%.

The sample calculation was performed using the formula for comparing paired groups with a quantitative variable as proposed by Miot [23], considering a 95% confidence level and 80% power. The sample size was 43 participants.

### 2.3. Intervention

The long-term progressive resistance training happened twice a week on nonconsecutive days and lasted one hour. In the 1^st^ and 2^nd^ weeks, the participants became familiar with the exercises and equipment and used a minimum load. Later, the 1-MR prediction test was applied. In the 3^rd^ and 4^th^ weeks, the participants trained with 60% of the 1-MR load; in the 5^th^ and 6^th^ weeks, they trained with 70% load; and in the 7^th^ and 8^th^ weeks, they trained with 80% load. From the 9th week, they trained with 85% of the 1-MR load until 19 weeks of training was completed. The number of repetitions suggested was 12-15 repetitions when the percentage of the 1-MR load was 60%, 10-12 repetitions for 70% load, and 6-8 repetitions for 80 and 85% of the 1-MR load [24, 25]. Adherence to resistance training was assessed using the 1-MR test applied before and after training [26].

The dietary advice was based on a list of foods rich in compounds with anti-inflammatory and antioxidant properties [10, 27–31]. The dietary advice was given to study participants by a nutritionist at the beginning of the training program in printed and verbal form. The advice was reinforced verbally every week. Adherence to dietary guidelines was assessed by calculating the total dietary antioxidant capacity (TACd) [32], using R24h [33] applied before and after dietary advice.

Participants were advised to increase the consumption of prebiotic foods (soluble fibers); antioxidants, such as polyphenols; and foods rich in omega 3, as well as to reduce the consumption of saturated fats and simple sugars. Therefore, the recommendation was to eat at least three fruits and two vegetables a day; natural spices (rosemary, turmeric, garlic, and onion), ginger, extra virgin olive oil, and whole grains (oats and flaxseed) once a day; and fish and chestnuts at least three times a week.

### 2.4. Anthropometric Measurements

Weight was measured using a portable Tanita® scale, a capacity of 150 kg, where the participant was weighed barefoot and wearing light clothing. Height was measured using a portable Sanny® anthropometer, spanning 115 to 210 cm [34]. The BMI was calculated using weight and height measurements to classify the nutritional status of the elderly, according to Lipschitz [35].

To measure the circumferences, a flexible and inelastic measuring tape was used. It was divided into centimeters and subdivided into millimeters (with a precision of 1 mm). The measurements followed Lohman et al. [34]. The AMAc was calculated using the equation by Heymsfield et al. [36] and the waist-hip ratio (WHR) was obtained by dividing WC by HC [37]. Skinfolds were measured using a Cescorf® adipometer with a sensitivity of 0.1 mm, reading range of 85 mm, and pressure of 10 g/mm [2, 34]. The BF percentage was estimated by summing four skinfold values, according to the equation of Durnin and Womersley [38].

### 2.5. Blood Collection and Analysis of Inflammatory Biomarkers

The blood collection was performed by a trained professional using intravenous puncture and vacuum system in the anterior area of the arm, in front of and below the elbow, where the medial and cephalic veins are. For the CRP analysis of the serum, a blood tube containing serum separation gel was used. For the analyses of IL-8, CCL-2, and leptin in the plasma, the collection was performed in a blood tube containing EDTA. The tubes remained at rest for 30 minutes. Then, they were centrifuged at 3,000 rpm for ten minutes. After separating the blood components, the aliquots were pipetted and stored.

The CRP dose evaluation was performed using the turbidimetric inhibition immunoassay and a specific kit for the Cobas Integra 400 Plus equipment (Roche®). The analyses of the biomarkers IL-8, CCL-2 (sensitivity from 8 to 1,000 pg/mL), and leptin (sensitivity from 63 to 4,000 pg/mL) were performed using the ELISA method and specific kits from PeproTech® following the manufacturer's protocol.

### 2.6. Statistical Analyses

Data were tabulated and assessed for normality by the Shapiro-Wilk test. The results were expressed as mean and standard deviation in the case of normal data or median (minimum and maximum) if nonparametric. Differences between paired groups were assessed using the paired *t*-test or Wilcoxon test (two groups), depending on data normality. Tests for normality and differences between groups were performed using the GraphPad Prism® software, version 6.0. For all analyses, a level of significance of 5% was adopted.

## 3. Results

**Figure 1:**
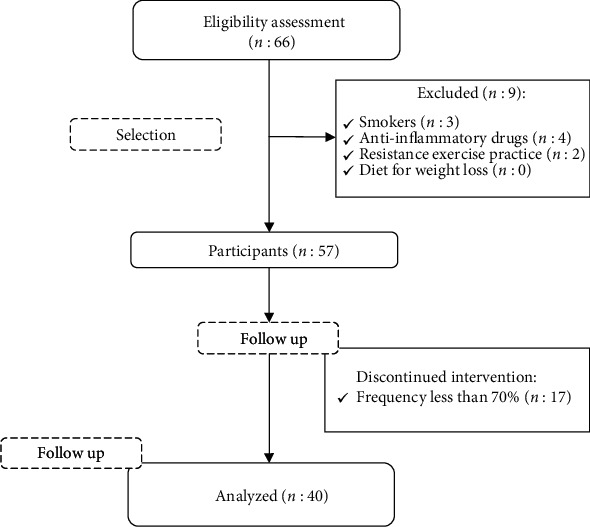
Flowchart of the present study.

The present study had the participation of 40 elderly people (Figure 1), with an average age of 63.90 ± 5.80 years, and among them, 26 (65%) were women.

**Figure 2:**
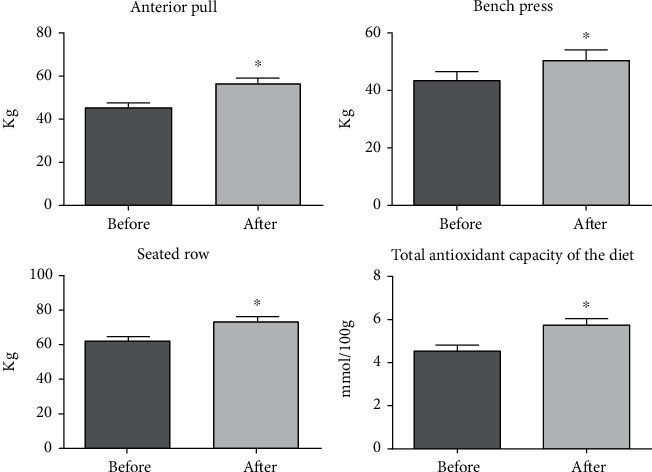
Evaluation of adherence to the intervention, performance data obtained by the 1-RM test, and antioxidant capacity of the diet calculated by R24h. ^∗^Difference between paired groups (paired *t*-test or Wilcoxon, *p* value < 0.05).

Regarding adherence to the intervention, there was an increase in performance in the exercises of anterior pull, bench press, and seated row, as assessed by the 1-MR test. There was an increase in TACd calculated by R24h (Figure 2), indicating that the participants adhered to the proposed intervention.

The intervention based on resistance training and dietary advice resulted in a reduction in WC, WHR, and BF percentage and in an increase in ArmC, AMAc, and CC (Table 1). Regarding inflammatory biomarkers, there was a reduction in plasma CCL-2 concentrations: 299.8 (54.4-950.7) pg/mL (before) and 229.9 (27.9-744.1) pg/mL (after) (*p* = 0.01), and in leptin:2,749 ± 693 pg/mL (before) and 2,405 ± 655 pg/mL (after) (*p* < 0.01).

**Figure 3:**
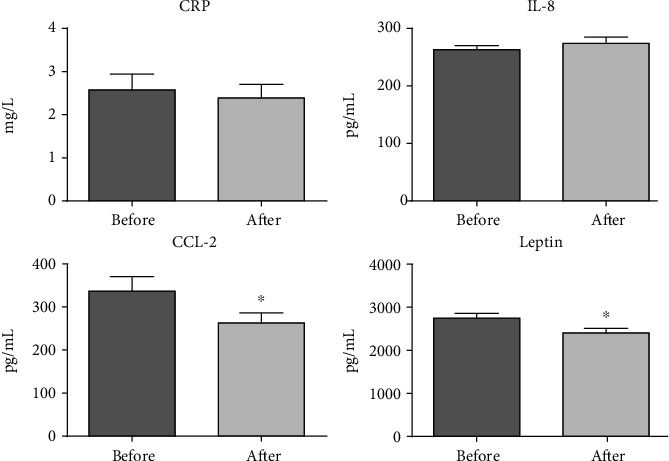
Results of inflammatory biomarkers (CRP, IL-8, CCL-2, and leptin) before and after the intervention. ^∗^Difference between paired groups (paired *t*-test or Wilcoxon, *p* value < 0.05).

However, there were no differences in CRP concentrations: 1.97 (0.27-8.06) mg/L (before) and 1.70 (0.15-7.17) mg/L (after) (*p* = 0.41), and IL-8: 259.6 (198.7-448.7) pg/mL (before) and 258.8 (169.1-491.3) pg/mL (after) (*p* = 0.97), according to Figure 3.

## 4. Discussion

Low-grade chronic inflammation is common with aging and is associated with an increased risk of developing diseases, such as NCDs and sarcopenia. Studies have indicated resistance training to help reduce chronic inflammation in the elderly population. However, the results are still controversial. In addition, existing studies have not addressed dietary advice focusing on foods rich in anti-inflammatory and antioxidant properties in conjunction with training [18–22].

In this scenario, the present study evaluated the effects of resistance training associated with dietary advice on chronic inflammation in elderly people living in the city of Ouro Preto, MG, Brazil. The results show a decrease in plasma concentrations of the inflammatory biomarkers CCL-2 and leptin and an improvement in body composition.

According to Pedersen and Febbraio [39], the muscle is an endocrine organ capable of producing and secreting cytokines, called myosins, in response to muscle contractions. Among myosins, IL-6 stimulates the expression of anti-inflammatory cytokines (IL-1ra and IL-10) and reduces the levels of circulating inflammatory biomarkers, such as IL-1*β*, TNF, and chemokines (CCL-2). Thus, skeletal muscle hypertrophy tends to increase the production of IL-6 by the muscle, which could explain the improvement in the inflammatory profile.

Regarding the reduction of plasma CCL-2 concentrations, the finding of this study can also be related to a decrease in visceral and subcutaneous fat since CCL-2 is also produced by the adipose tissue, as well as leptin. Other studies have shown that resistance training is effective in reducing adiposity due to increased metabolic rates at rest, improved insulin sensitivity, and increased sympathetic activity, which reduces visceral fat storage [40, 18, 41–46].

However, there are still few studies evaluating the chronic effects of resistance training on the CCL-2 biomarker in the elderly. Supposedly, because aerobic exercises have a greater capacity for lipid oxidation due to the increase in the number of mitochondria [40]. In this scenario, Ihalainen et al. [47] corroborate the present study by reporting a decrease in body fat and in the inflammatory biomarkers CCL-2 and leptin after 24 weeks of combined training (resistance and aerobic exercises) in adults. Similarly, Leggate et al. [48] showed a reduction in WC and CCL-2 in obese individuals after high-intensity exercises.

In the elderly, Kelly et al. [49] showed that 12 weeks of aerobic training reduced CCL-2 levels in obese elderly people. On the other hand, Ogawa et al. [50] did not observe changes in the plasma concentrations of CCL-2 in elderly women after 12 weeks of resistance training (once a week). Considering that the aforementioned studies have emphasized that exercise intensity is relevant to change CCL-2 levels, the progression of intensity (65 to 85% of 1-MR) in the present study may have positively influenced the response of this cytokine.

Another possible mechanism for reducing CCL-2 concentrations involves the production of reactive oxygen species. Resistance training is known to increase glucose uptake in muscles, reducing plasma glucose concentration and consequently the production of reactive oxygen species, which inhibits the NF-*κ*B pathway, responsible for expressing genes that encode proinflammatory cytokines such as CCL-2 [51, 49, 39, 52, 53]. In addition, adaptations resulting from chronic exercise lead to the reduction of reactive oxygen species by increasing the activity of antioxidant enzymes [54, 55, 21, 56].

The reduction in plasma leptin concentrations may also be associated with an improvement in body composition. Pedersen and Febbraio [57] described that myosins produced by the increase in muscle mass neutralize the production of adipokines due to the increase in lipid oxidation in the adipose tissue. Still regarding this scenario, other authors reported that leptin is produced predominantly by white adipose tissue, and its plasma concentration is proportional to the amount of body fat [51, 58, 59]. Thus, the increase in muscle mass and the reduction in body fat, as evidenced in the present study, affected leptin levels in the elderly.

In this scenario, Balducci et al. [60] showed that combined training (resistance and aerobic exercises) for one year, whose intensity of resistance is 80% of 1-MR and the frequency is two times a week, reduces leptin levels in diabetic and obese elderly. Prestes et al. [61] showed a reduction in plasma leptin concentrations in elderly women after 16 weeks of resistance training with a frequency of two times a week.

The changes found can also be justified by better eating habits promoted by dietary advice and evidenced by the increase in TACd. The diet may have foods rich in compounds with anti-inflammatory and antioxidant properties, such as polyphenols, prebiotics, and omega 3 fatty acids [5, 62]. These compounds modulate the Nrf2 signaling pathway, increasing the expression of antioxidant enzymes and reducing the production of reactive oxygen species [5, 63, 51].

Studies have addressed that bioactive compounds present in food can improve leptin resistance by acting in the recovery of signaling between leptin and neurotransmitters and in the transport of this adipokine to the blood-brain barrier [64, 58].

Corroborating the results found in the present study, Shah et al. [65] also observed a reduction in plasma leptin concentrations in obese elderly people after 24 weeks of combined training (resistance and aerobic exercises) associated with a proper diet. In that study, the intensity of resistance exercises was between 65 and 80% of 1-MR, similar as the methodology applied in the present research.

In the present study, the serum levels of CRP did not change, which can be justified by the fact that the elderly had normal values (less than 2 mg/L) before the intervention. According to Gaesser et al. [66], CRP reduction is more common in individuals with high levels of this biomarker before interventions. There were also no differences in plasma IL-8 concentrations. Corroborating this finding, Buford et al. [67] suggested that IL-8 has a local effect on the muscle after resistance training and plasma changes tend not to occur.

The WHO [68] recommends to the elderly population at least 150 minutes of moderate aerobic exercise per week for the control of CNCD, in which chronic low-grade inflammation is directly involved. However, the role of resisted exercise is still little studied [40]. Fragala et al. [24] and Sardeli et al. [69] reported that a resistance exercise program lasting 12 weeks or more, with at least eight exercises and a frequency of three times a week, is effective in reducing proinflammatory cytokines in the elderly.

Thus, this study, using new biomarkers, elucidates the use of relatively simple interventions in the planning of assistance programs for the elderly, including resistance exercises and dietary advice, as a way to reduce health risks.

The absence of a control group to identify the isolated effects of resistance training and dietary advice is a limitation of the study. However, the aim of this study was precisely to demonstrate the synergistic effect of both strategies in reducing low-grade chronic inflammation. The combination of both strategies has been widely reported as more effective than isolated actions regarding physical exercises and changes in eating habits [49, 70–72]. In addition, the study design and adherence to the intervention are strengths of this study.

## 5. Conclusion

The present study shows that long-term progressive resistance training associated with dietary advice for 19 weeks is effective in reducing inflammatory biomarkers (CCL-2 and leptin). In addition, there is a reduction in the anthropometric parameters WC and BF percentage and an increase in ArmC, CC, and AMAc, evidencing a reduction in body fat and an increase in muscle mass. Resistance training associated with dietary advice can contribute to a healthy aging due to observed improvements in body composition and in the inflammatory profile of the elderly.

## Figures and Tables

**Table 1:** Results regarding anthropometric variables before and after the intervention.

*n*: 40				
Variable	Before	After	*p* value	Δ
Weight (kg)	72.08 ± 13.78	71.95 ± 13.26	0.82	0.13
BMI (kg/^m2)^	27.68 ± 4.52	27.62 ± 4.21	0.78	0.06
WC (cm)	89.43 ± 10.05	86.53 ± 10.02	<0.01	2.90
HC (cm)	99.10 (85.40-119.20)	101 (86.80-121)	0.40	-1.90
WHR	0.89 ± 0.08	0.86 ± 0.08	<0.01	0.04
AC (cm)	93.80 ± 12.73	93.22 ± 12.72	0.55	0.58
ArmC (cm)	30.31 ± 4.03	31.00 ± 3.62	0.04	-0.69
AMAc (cm^2^)	37.74 ± 18.42	44.89 ± 17.57	<0.01	-7.15
CC (cm)	36 (26.80-42.50)	37.50 (30.30-59.80)	<0.01	-1.50
BF (%)	38.69 ± 7.00	34.77 ± 6.13	<0.01	3.75

Δ: delta (initial-final); BMI: body mass index; WC: waist circumference; HC: hip circumference; WHR: waist-to-hip ratio; AC: abdominal circumference; ArmC: arm circumference; AMAc: corrected arm muscle area; CC: calf circumference; BF (%): percentage of body fat. ^∗^Difference between paired groups (paired *t*-test or Wilcoxon, *p* value < 0.05).

## Data Availability

This manuscript was prepared based on the Postgraduate in Health and Nutrition that is available at the Institutional Repository of the Federal University of Ouro Preto (http://www.repositorio.ufop.br/handle/123456789/12340).

## References

[1] Mello A. C., Carvalho M. S., Alves L. C., Gomes V. P., Engstrom E. M. (2017). Food consumption and anthropometry related to the frailty syndrome in low-income community-living elderly in a large city. *Cadernos de Saúde Pública*.

[2] Veras R., Oliveira M. (2016). Care pathway for the elderly: detailing the model. *Revista Brasileira de Geriatria e Gerontologia*.

[3] Tavares E. L., Santos D. M. ., Ferreira A. A., Menezes M. F. G. . (2015). Avaliação nutricional de idosos: desafios da atualidade. *Revista Brasileira de Geriatria e Gerontologia*.

[4] Martins R. A., Veríssimo M. T., Coelho e Silva M. J., Cumming S. P., Teixeira A. M. (2010). Effects of aerobic and strength-based training on metabolic health indicators in older adults. *Lipids in Health and Disease*.

[5] Calder P. C., Bosco N., Bourdet-Sicard R. (2017). Health relevance of the modification of low grade inflammation in ageing (inflammageing) and the role of nutrition. *Ageing Research Reviews*.

[6] Pera A., Campos C., López N. (2015). Immunosenescence: implications for response to infection and vaccination in older people. *Maturitas*.

[7] Calçada D., Vianello D., Giampieri E. (2014). The role of low-grade inflammation and metabolic flexibility in aging and nutritional modulation thereof: a systems biology approach. *Mechanisms of Ageing and Development*.

[8] Michaud M., Balardy L., Moulis G. (2013). Proinflammatory cytokines, aging, and age-related diseases. *Journal of the American Medical Directors Association*.

[9] Palmer D. B. (2013). The effect of age on thymic function. *Frontiers in Immunology*.

[10] Custodero C., Mankowski R. T., Lee S. A. (2018). Evidence-based nutritional and pharmacological interventions targeting chronic low-grade inflammation in middle-age and older adults: a systematic review and meta-analysis. *Ageing Research Reviews*.

[11] Ribeiro A. S., Schoenfeld B. J., Souza M. F. (2016). Traditional and pyramidal resistance training systems improve muscle quality and metabolic biomarkers in older women: a randomized crossover study. *Experimental Gerontology*.

[12] Beavers K. M., Brinkley T. E., Nicklas B. J. (2010). Effect of exercise training on chronic inflammation. *Clinica Chimica Acta*.

[13] Scheele C., Nielsen S., Pedersen B. K. (2009). ROS and myokines promote muscle adaptation to exercise. *Trends in Endocrinology & Metabolism*.

[14] Starkie R., Ostrowski S. R., Jauffred S., Febbraio M., Pedersen B. K. (2003). Exercise and IL-6 infusion inhibit endotoxin-induced TNF‐*α* production in humans. *The FASEB Journal*.

[15] Toni M., Massimino M. L., de Mario A., Angiulli E., Spisni E. (2017). Metal dyshomeostasis and their pathological role in prion and prion-like diseases: the basis for a nutritional approach. *Frontiers in Neuroscience*.

[16] Schwingshackl L., Hoffmann G. (2014). Mediterranean dietary pattern, inflammation and endothelial function: a systematic review and meta-analysis of intervention trials. *Nutrition, Metabolism and Cardiovascular Diseases*.

[17] Calder P. C., Ahluwalia N., Brouns F. (2011). Dietary factors and low-grade inflammation in relation to overweight and obesity. *British Journal of Nutrition*.

[18] Tomeleri C. M., Souza M. F., Burini R. C. (2018). Resistance training reduces metabolic syndrome and inflammatory markers in older women: a randomized controlled trial. *Journal of Diabetes*.

[19] Tomeleri C. M., Ribeiro A. S., Souza M. F. (2016). Resistance training improves inflammatory level, lipid and glycemic profiles in obese older women: a randomized controlled trial. *Experimental Gerontology*.

[20] Mavros Y., Kay S., Simpson K. A. (2014). Reductions in C-reactive protein in older adults with type 2 diabetes are related to improvements in body composition following a randomized controlled trial of resistance training. *Journal of Cachexia, Sarcopenia and Muscle*.

[21] Beltran Valls M. R., Dimauro I., Brunelli A. (2014). Explosive type of moderate-resistance training induces functional, cardiovascular, and molecular adaptations in the elderly. *Age*.

[22] Phillips M. D., Flynn M. G., Mcfarlin B. K., Stewart L. K., Timmerman K. L. (2010). Resistance training at eight-repetition maximum reduces the inflammatory milieu in elderly women. *Medicine & Science in Sports & Exercise*.

[23] Miot H. A. (2011). Tamanho da amostra em estudos clínicos e experimentais. *Jornal Vascular Brasileiro*.

[24] Fragala M. S., Cadore E. L., Dorgo S. (2019). Resistance training for older adults: position statement from the national strength and conditioning association. *The Journal of Strength & Conditioning Research*.

[25] Chodzko-Zajko W. J., Proctor D. N., Fiatarone Singh M. A. (2009). Exercise and physical activity for older adults. *Medicine & Science in Sports & Exercise*.

[26] Brzycki M. (1993). Strength testing—predicting a one-rep max from reps-to-fatigue. *Journal of Physical Education, Recreation & Dance*.

[27] Lee A., Thurnham D. I., Chopra M. (2000). Consumption of tomato products with olive oil but not sunflower oil increases the antioxidant activity of plasma. *Free Radical Biology and Medicine*.

[28] Caballero-Gutiérrez L., Gonzáles G. F. (2016). Alimentos con efecto antiinflamatorio. *Acta Medica Peruana*.

[29] Tabung F. K., Steck S. E., Zhang J. (2015). Construct validation of the dietary inflammatory index among postmenopausal women. *Annals of Epidemiology*.

[30] Kishimoto Y., Tani M., Kondo K. (2013). Pleiotropic preventive effects of dietary polyphenols in cardiovascular diseases. *European Journal of Clinical Nutrition*.

[31] Ruiz-Núñez B., Pruimboom L., Dijck-Brouwer D. A. J., Muskiet F. A. J. (2013). Lifestyle and nutritional imbalances associated with Western diseases: causes and consequences of chronic systemic low-grade inflammation in an evolutionary context. *Journal of Nutritional Biochemistry*.

[32] Carlsen M. H., Halvorsen B. L., Holte K. (2010). The total antioxidant content of more than 3100 foods, beverages, spices, herbs and supplements used worldwide. *Nutrition Journal*.

[33] Fisberg R. M., Marchioni D. M. L., Previdelli A. N. (2012). Manual de avaliação do consumo alimentar em estudos populacionais: a experiência do inquérito de saúde em São Paulo (ISA). http://colecoes.sibi.usp.br/fsp/items/show/2419.

[34] Lohman T. G., Roche A. F., Martorell R. (1988). *Anthropometric standardization reference manual*.

[35] Lipschitz D. A. (1994). Screening for nutritional status in the elderly. *Primary Care*.

[36] Heymsfield S. B., McManus C., Smith J., Stevens V., Nixon D. W. (1982). Anthropometric measurement of muscle mass: revised equations for calculating bone-free arm muscle area. *The American Journal of Clinical Nutrition*.

[37] World Health Organization (1998). *Obesity: preventing and managing the global epidemic*.

[38] Durnin J. V. G. A., Womersley J. (1974). Body fat assessed from total body density and its estimation from skinfold thickness: measurements on 481 men and women aged from 16 to 72 years. *British Journal of Nutrition*.

[39] Pedersen B. K., Febbraio M. A. (2008). Muscle as an endocrine organ: focus on muscle-derived interleukin-6. *Physiological Reviews*.

[40] Mcleod J. C., Stokes T., Phillips S. M. (2019). Resistance exercise training as a primary countermeasure to age-related chronic disease. *Frontiers in Physiology*.

[41] Westcott W. L. (2012). Resistance training is medicine. *Current Sports Medicine Reports*.

[42] Hurley B. F., Hanson E. D., Sheaff A. K. (2011). Strength training as a countermeasure to aging muscle and chronic disease. *Sports Medicine*.

[43] Shaw C. S., Clark J., Wagenmakers A. J. M. (2010). The effect of exercise and nutrition on intramuscular fat metabolism and insulin sensitivity. *Annual Review of Nutrition*.

[44] Ibanez J., Izquierdo M., Arguelles I. (2005). Twice-weekly progressive resistance training decreases abdominal fat and improves insulin sensitivity in older men with type 2 diabetes. *Diabetes Care*.

[45] Hunter G. R., Wetzstein C. J., Fields D. A., Brown A., Bamman M. M. (2000). Resistance training increases total energy expenditure and free-living physical activity in older adults. *Journal of Applied Physiology*.

[46] Campbell W., Crim M. C., Young V. R., Evans W. J. (1994). Increased energy requirements and changes in body composition with resistance training in older adults. *The American Journal of Clinical Nutrition*.

[47] Ihalainen J. K., Schumann M., Eklund D. (2018). Combined aerobic and resistance training decreases inflammation markers in healthy men. *Scandinavian Journal of Medicine & Science in Sports*.

[48] Leggate M., Carter W. G., Evans M. J. C., Vennard R. A., Sribala-Sundaram S., Nimmo M. A. (2012). Determination of inflammatory and prominent proteomic changes in plasma and adipose tissue after high-intensity intermittent training in overweight and obese males. *Journal of Applied Physiology*.

[49] Kelly K. R., Haus J. M., Solomon T. P. J. (2011). A low-glycemic index diet and exercise intervention reduces TNF*α* in isolated mononuclear cells of older, obese adults. *The Journal of Nutrition*.

[50] Ogawa K., Sanada K., Machida S., Okutsu M., Suzuki K. (2010). Resistance exercise training-induced muscle hypertrophy was associated with reduction of inflammatory markers in elderly women. *Mediators of inflammation*.

[51] Cominetti C., Rogero M. M., Horst M. A. (2017). *Genômica nutricional: dos fundamentos à nutrição molecular*.

[52] Bruun J. M., Lihn A. S., Pedersen S. B., Richelsen B. (2005). Monocyte chemoattractant protein-1 release is higher in visceral than subcutaneous human adipose tissue (AT): implication of macrophages resident in the AT. *The Journal of Clinical Endocrinology & Metabolism*.

[53] Murao K., Imachi H., Momoi A. (1999). Thiazolidinedione inhibits the production of monocyte chemoattractant protein-1 in cytokine-treated human vascular endothelial cells. *FEBS Letters*.

[54] Azizbeigi K., Azarbayjani M. A., Atashak S., Stannard S. R. (2015). Effect of moderate and high resistance training intensity on indices of inflammatory and oxidative stress. *Research in Sports Medicine*.

[55] Padilha C. S., Ribeiro A. S., Fleck S. J. (2015). Effect of resistance training with different frequencies and detraining on muscular strength and oxidative stress biomarkers in older women. *Age*.

[56] Gomes E. C., Silva A. N., Oliveira M. R. . (2012). Oxidants, antioxidants, and the beneficial roles of exercise-induced production of reactive species. *Oxidative Medicine and Cellular Longevity*.

[57] Pedersen B. K., Febbraio M. A. (2012). Muscles, exercise and obesity: skeletal muscle as a secretory organ. *Nature Reviews Endocrinology*.

[58] Aragonès G., Ardid-Ruiz A., Ibars M., Suárez M., Bladé C. (2016). Modulation of leptin resistance by food compounds. *Molecular Nutrition & Food Research*.

[59] Martins M. C., Lima Faleiro L., Fonseca A. (2012). Relação entre a leptina, a massa corporal e a síndrome metabólica numa amostra da população adulta. *Revista Portuguesa de Cardiologia*.

[60] Balducci S., Zanuso S., Nicolucci A. (2010). Anti-inflammatory effect of exercise training in subjects with type 2 diabetes and the metabolic syndrome is dependent on exercise modalities and independent of weight loss. *Nutrition, Metabolism and Cardiovascular Diseases*.

[61] Prestes J., Shiguemoto G., Botero J. P. (2009). Effects of resistance training on resistin, leptin, cytokines, and muscle force in elderly post-menopausal women. *Journal of Sports Sciences*.

[62] Volp A. C. P., Bressan J., Hermsdorff H. H. M., Zulet M. Á., Martínez J. A. (2010). Efeitos antioxidantes do selênio e seu elo com a inflamação e síndrome metabólica. *Revista de Nutrição*.

[63] Yu B. P., Chung H. Y. (2006). Adaptive mechanisms to oxidative stress during aging. *Mechanisms of Ageing and Development*.

[64] Ardid-Ruiz A., Harazin A., Barna L. (2020). The effects of Vitis vinifera L. phenolic compounds on a blood-brain barrier culture model: expression of leptin receptors and protection against cytokine-induced damage. *Journal of Ethnopharmacology*.

[65] Shah K., Armamento-Villareal R., Parimi N. (2011). Exercise training in obese older adults prevents increase in bone turnover and attenuates decrease in hip bone mineral density induced by weight loss despite decline in bone-active hormones. *Journal of Bone and Mineral Research*.

[66] Gaesser G. A., Angadi S. S., Ryan D. M., Johnston C. S. (2011). Lifestyle measures to reduce inflammation. *American Journal of Lifestyle Medicine*.

[67] Buford T. W., Cooke M. B., Willoughby D. S. (2009). Resistance exercise-induced changes of inflammatory gene expression within human skeletal muscle. *European Journal of Applied Physiology*.

[68] World Health Organization (2011). Global recommendations on physical activity for health. http://www.who.int/dietphysicalactivity/publications/9789241599979/en/.

[69] Sardeli A. V., Tomeleri C. M., Cyrino E. S., Fernhall B., Cavaglieri C. R., Chacon-Mikahil M. P. T. (2018). Effect of resistance training on inflammatory markers of older adults: a meta-analysis. *Experimental Gerontology*.

[70] Giannopoulou I., Fernhall B., Carhart R. (2005). Effects of diet and/or exercise on the adipocytokine and inflammatory cytokine levels of postmenopausal women with type 2 diabetes. *Metabolism*.

[71] Dean E., Gormsen Hansen R. (2012). Prescribing optimal nutrition and physical activity as “first-line” interventions for best practice management of chronic low-grade inflammation associated with osteoarthritis: evidence synthesis. *Arthritis*.

[72] Corsetto P. A., Montorfano G., Klersy C. (2019). Fatty acid profile and antioxidant status fingerprint in sarcopenic elderly patients: role of diet and exercise. *Nutrients*.

